# Inflammatory Breast Cancer: High Incidence of Detection of Mixed Human Cytomegalovirus Genotypes Associated with Disease Pathogenesis

**DOI:** 10.3389/fonc.2014.00246

**Published:** 2014-09-11

**Authors:** Hossam Taha Mohamed, Mohamed El-Shinawi, M. Akram Nouh, Abdel-Rahman Bashtar, Elsayed Tarek Elsayed, Robert J. Schneider, Mona Mostafa Mohamed

**Affiliations:** ^1^Department of Zoology, Faculty of Science, Cairo University, Giza, Egypt; ^2^Department of General Surgery, Faculty of Medicine, Ain Shams University, Cairo, Egypt; ^3^Department of Pathology, National Cancer Institute, Cairo University, Giza, Egypt; ^4^Department of Botany, Faculty of Science, Cairo University, Giza, Egypt; ^5^Department of Microbiology, School of Medicine, New York University, New York, NY, USA

**Keywords:** human cytomegalovirus, glycoproteins, inflammatory breast neoplasms, UL55, UL73, lymphovascular invasion, metastasis

## Abstract

Inflammatory breast cancer (IBC) is a highly metastatic, aggressive, and fatal form of breast cancer. Patients presenting with IBC are characterized by a high number of axillary lymph node metastases. Recently, we found that IBC carcinoma tissues contain significantly higher levels of human cytomegalovirus (HCMV) DNA compared to other breast cancer tissues that may regulate cell signaling pathways. In fact, HCMV pathogenesis and clinical outcome can be statistically associated with multiple HCMV genotypes within IBC. Thus, in the present study, we established the incidence and types of HCMV genotypes present in carcinoma tissues of infected non-IBC versus IBC patients. We also assessed the correlation between detection of mixed genotypes of HCMV and disease progression. Genotyping of HCMV in carcinoma tissues revealed that glycoprotein B (gB)-1 and glycoprotein N (gN)-1 were the most prevalent HCMV genotypes in both non-IBC and IBC patients with no significant difference between patients groups. IBC carcinoma tissues, however, showed statistically significant higher incidence of detection of the gN-3b genotype compared to non-IBC patients. The incidence of detection of mixed genotypes of gB showed that gB-1 + gB-3 was statistically significantly higher in IBC than non-IBC patients. Similarly, the incidence of detection of mixed genotypes of gN showed that gN-1 + gN-3b and gN-3 + gN-4b/c were statistically significant higher in the carcinoma tissues of IBC than non-IBC. Mixed presence of different HCMV genotypes was found to be significantly correlated with the number of metastatic lymph nodes in non-IBC but not in IBC patients. In IBC, detection of mixed HCMV different genotypes significantly correlates with lymphovascular invasion and formation of dermal lymphatic emboli, which was not found in non-IBC patients.

## Introduction

Breast cancer is considered the most common malignancy and a leading cause of death in women worldwide (1). Many risk factors including age, sex, obesity, estrogen level, and family history of breast cancer are contributors to increased breast cancer incidence (2). Studies suggest that viral infection maybe a risk factor in breast cancer (3, 4). For instance, a link between certain viral infections and breast cancer incidence is suspected for Epstein–Barr virus (EBV) (5), human papillomavirus (HPV) (6), mouse mammary tumor virus (MMTV) (7), and human cytomegalovirus (HCMV) (8), although causality remains unproven. HCMV has been detected in newly diagnosed (9) and metastatic breast cancer patients (10). Furthermore, HCMV proteins and DNA have been detected in breast ductal carcinoma *in situ* (DCIS) and infiltrating ductal carcinoma tissues, possibly suggesting a causal association of HCMV in breast carcinogenesis (8). The ability of HCMV to infect a wide variety of cells constituting the tumor microenvironment, such as monocytes/macrophages, fibroblasts, and endothelial cells, implicates a variety and possible mechanisms for HCMV in cancer pathogenesis (11–13).

During one’s lifetime, infection with several HCMV strains may occur (14). The virulence among different HCMV strains may be a crucial factor in the manifestation of HCMV-associated disease (15). It should be noted that genetic variation exists among HCMV genes that are implicated in host cell penetration, tissue tropism, or replication, which may influence the virulence of different HCMV strains (16). The severity of HCMV pathogenicity differs depending on genetic variability within viral genes (17). Among the most variable HCMV genes are the viral envelope glycoproteins such as *UL55* that encodes glycoprotein B (gB) (18), and *UL73* that encodes glycoprotein N (gN) (19). The gB genotype includes five genotypes (gB-1–gB-5) (20), while gN shows four genotypes (gN-1, gN-2, gN-3, and gN-4). The gN-3 genotype includes two subgenotypes (a and b) and gN-4 includes three subgenotypes (a, b, and c) (21).

Human cytomegalovirus gB is the major component of the virus envelope; it plays an essential role in viral entry into the cell by binding to membranous beta-1 integrin via its gB disintegrin-like domain, which mediates virus entry to the host cell and cell to cell virus transmission (22). Virus binding to the cell surface induces the phosphorylation of β1 and β3 integrins’ intracellular domains (23). The gN moiety is covalently linked through disulfide bonds with glycoprotein M to form gM/gN complex (24). It is assumed that gN alone or the gM/gN complex is implicated in virus entry and spread, and is essential for HCMV replication (25). Recently, it was found that the glycosylation of gN protects the virus from neutralizing antibodies (26).

Human cytomegalovirus cellular entry activates intracellular signaling pathways such as focal adhesion kinase (FAK) (23), mitogen-activated protein kinase (MAPK) (27), and phosphatidylinositol-3-OH kinase (PI3-K) (28) all of which are known to play key roles in cancer progression (29–31). In addition, they played the role of HCMV gB in cell adhesion and signaling capabilities (22, 23). HCMV infection is involved in cancer pathogens via modulation of apoptosis, cell migration, and angiogenesis (32). In fact, mixed infections with different genotypes of HCMV were detected in healthy (14) and immuno-suppressed patients, including patients undergoing lung transplant (33) and AIDS patients (34). In addition, mixed genotypes infection was found to be associated with increased morbidity and mortality of solid organ transplant patients (35, 36).

We are interested in the biology of inflammatory breast cancer (IBC), an aggressive and fatal form of breast cancer that occurs at increased incidence among young women (37–41). Our recent results found, for the first time, that their increased tumor-associated monocytes/macrophages (TAM/M) and HCMV infection in the biology of IBC disease (42, 43). We found that cytokines secreted by IBC TAM/M such as IL-10 and IL-8 may be associated with HCMV infection. We screened for HCMV infection in IBC versus non-IBC patients. Serological diagnosis indicates that HCMV antibody titer was higher in IBC versus non-IBC. Furthermore, nested PCR results revealed that HCMV-DNA was detected in carcinoma tissues of IBC and not in adjacent non-carcinoma tissues, which was statistically significant compared to the non-IBC patient group. Moreover, we found that the expression and activation (phosphorylation) of NF-κB/p65 signaling molecules are enhanced in HCMV-infected IBC cancer tissues compared to non-IBC tumors. These data all suggest a possible pathogenic role of HCMV in IBC at a greater level than in non-IBC carcinomas (43).

In the present study, we established the incidence of single and mixed HCMV gB and gN genotypes DNA in carcinoma tissues of non-IBC versus IBC patients. Furthermore, we assessed the correlation between mixed genotypes and major pathological properties associated with breast cancer disease progression.

## Materials and Methods

### Patients and samples

For participation of patients in this study, Institutional Review Board (IRB) approval was obtained from the ethics committee of Ain-Shams University. Each patient before participation provided a signed consent form including approval for publication of results. Patients visiting the breast clinic of Ain-Shams University hospitals and diagnosed with breast cancer by clinical examination, mammography, ultrasound, and tru-cut biopsy (using a needle for manual culture of the breast tissues) were recruited to the present study (42). In addition to the previous criteria, breast cancer patients presenting with swollen breast, edema, skin orange peel (pead’orange), both skin and core biopsies showed infiltration of dermal lymphatics by carcinoma cells and the presence of dermal tumor emboli, as we described previously (40) and were diagnosed as IBC patients. By applying these criteria, a total of 147 patients (99 non-IBC and 48 IBC) with HCMV-DNA positive carcinoma tissue samples (43) were recruited in the present study during the period of January 2010–September 2013.

### Multiplex PCR assay for HCMV glycoproteins B and N genotypes

We extracted DNA from fresh breast carcinoma tissues obtained during modified radical mastectomy using GeneJET™ Genomic DNA purification Kit (Thermo scientific, Waltham, MA, USA). Experimental procedures were carried out as described in the kit guidelines. The oligonucleotide primers used for nested and multiplex PCR were commercially synthesized (Invitrogen, Carlsbad, CA, USA) and are listed in Table 1. Detection of HCMV-DNA from breast carcinoma tissue samples was carried out using nested PCR with two sets of primers specific for the fourth exon of the HCMV Immediate Early (IE) gene as we described previously (43). Genotyping of HCMV gB and gN was performed by multiplex PCR using a mixture of specific primers to each of gB and gN genotypes. For detection of different gB genotypes, nested multiplex PCR was performed with two external primers and five upstream inner primers specific for each gB genotype (gB-1, gB-2, gB-3, gb-4, and gB-5) and a single downstream primer as described elsewhere (44). The first round of the nested multiplex PCR was carried out in a 25 μl total volume using 1 μl of each external upstream and downstream primers (10 pmol∖ml), 3 μl of DNA, containing a maximum of 250 ng DNA from fresh breast carcinoma tissue, 12.5 μl of EmeraldAmp^®^ MAX PCR green master mix (Takara, Dalian, China), and 7.5 μl of free RNase water. The PCR thermal profile started with an initial denaturation at 94°C for 5 min, followed by 35 cycles at 94°C for 45 s, 60°C for 1 min, and 72°C for 45 s, followed by terminal extension at 72°C for 10 min. The second round of PCR was performed using 5 μl of the first reaction as DNA template and an equimolar mixture of 10 pmol of each inner primer in a 25 μl total volume. Reaction was carried out under conditions identical to those used in the first round, but the annealing temperature was 58°C instead of 60°C. For detection of gN genotypes, multiplex PCR was done with five specific upstream primers for each gN genotypes (gN-1, gN-2, gN-3b, gN-4a, and gN-4b/c) and single downstream primers as described elsewhere (45). The reaction was carried out with an equimolar mixture of 10 pmol of the five upstream primers and the single downstream primer in a 25 μl total volume with a thermal profile including an initial denaturation at 94°C for 5 min, followed by 35 cycles of 94°C for 45 s, 60°C for 1 min, and 72°C for 45 s, followed by terminal extension at 72°C for 10 min.

**Table 1:** **Primers sequences, target gene, and expected amplicon length used in viral screening**.

Gene	Target	Direction	Sequence	Product (bp)
*UL55*	Entire gene amplification	F	GGGAGCCGCACCGACCTT	2782
		R	GGTCACGCCGCCGCTCAG	
	Nested multiplex PCR	F (external)	TTTGGAGAAAACGCCGAC	751
		R (external)	GCGGCAATCGGTTTGTTGTA	
		F1 (inner)	ATGACCGCCACTTTCTTATC	420
		F2 (inner)	TTCCGACTTTGGAAGACCCAAC	613
		F3 (inner)	TAGCTCCGGTGTGAACTCC′	190
		F4 (inner)	ACCATTCGTTCCGAAGCCGAGGAGTCA	465
		F5 (inner)	TACCCTATCGCTGGAGAAC	139
		R (inner)	GTTGATCCACACACCAGGC	
*UL73*	Entire gene amplification	F	TGGTGTGATGGAGTGGAAC	421
		R	TAGCCTTTGGTGGTGGTTGC	
	Multiplex PCR	F1	TTCTGCTAGCGTATCAACTACC	283
		F2	AGTGCAAAACACTGG TGCT	380
		F-3b	CACAACCACATTAACGAGT	214
		F-4a	CAACAATACGTCGACT GCTAGCACAC	325
		F-4b/c	GACAACTAGT ACAACTACGGTGACAA	244
		R	GACATTGCTGCTTCCAGAA	

*Source of the primers*.

*UL55 entire gene amplification primers are described by Meyer-Konig et al. (14)*.

*UL55 nested multiplex PCR primers are described by Tarrago et al. (44)*.

*UL73 entire gene amplification primers are described by Yan et al. (46)*.

*UL73 multiplex PCR primers are described by Pignatelli et al. (45)*.

### Amplification of gB and gN entire genes

For gB and gN sequencing, the entire *gB* gene (2782 bp) was amplified in a 50 μl total volume containing 1 μl of each upstream and downstream primers (10 pmol∖ml), 5 μl of the DNA containing a maximum 300 ng DNA from fresh breast carcinoma tissue, 25 μl of EmeraldAmp^®^ MAX PCR green master mix, and 18 μl of free RNase water. The thermal profile included an initial denaturation at 94°C for 5 min, followed by 35 cycles of 94°C for 45 s, 60°C for 1 min, and 72°C for 2 min, followed by terminal extension at 72°C for 10 min as described by Meyer-Konig et al. (14). Similarly, the entire *gN* gene (418 bp) was amplified, the thermal profile included an initial denaturation at 94°C for 5 min, followed by 35 cycles of 94°C for 45 s, 53°C for 1 min, and 72°C for 1 min, followed by terminal extension at 72°C for 10 min as described by Yan et al. (46).

### Agarose gel electrophoresis of PCR products

Amplified PCR products were visualized on 2% agarose gels (Bio Basic Inc., Canada), stained with ethidium bromide, and photographed by the GBOX-F3 gel documentation system Syngene (Syngene, MD, USA). All PCR reactions were conducted using sterilized tubes and tips in a biosafety hood that was not previously exposed to any virology work.

### DNA sequencing of PCR products

To assure the results of HCMV genotyping, the entire gB and gN genes were sequenced. We purified 18 randomly selected agarose gel PCR products (9 non-IBC and 9 IBC) using GeneJET™ Gel Extraction Kit (Thermo scientific, Waltham, MA, USA). Purified PCR products were commercially sequenced at GATC-Biotech (Konstanz, Germany). Single-pass sequencing was performed on each sample using the same upstream and downstream primers used in the amplification of the entire gB and gN genes. Confirmation of viral DNA sequences and multiple alignments were performed using NCBI Blast search analysis.

### Statistical analysis

Data were expressed as mean ± SD. Statistical differences or comparison between two groups was assessed by Student’s *t*-test and Fisher’s exact test. Correlation was assessed by Pearson’s correlation coefficient using SPSS 18.0 software (43).

## Results

### Clinical and pathological characterization of non-IBC versus IBC patients

Clinical and pathological characterization of patients who participated in the present study is described in Table 2. The status of lymph node metastasis showed that IBC patients had a significantly (*p* = 0.001) higher number of metastatic lymph nodes compared to non-IBC patients consistent with the typically higher stage at presentation. In addition, the status of lymphovascular invasion and presence of dermal lymphatic emboli in IBC patients was significantly higher (*p* = 0.001) than in non-IBC patients, again consistent with determination of IBC.

**Table 2:** **Clinical and pathological characterization of non-IBC versus IBC patients**.

Characteristic	Non-IBC (*N* = 99)	IBC (*N* = 48)	*p* Value
Age (years)
Range	27–78	29–72	0.152^a^
Mean ± SD	52.35 ± 11	49.52 ± 11.59	
Tumor size (cm)
Mean ± SD	4.6 ± 3.5	5.73 ± 2.71	0.001*^a^
≤4	63 (63.6%)	17 (35.4%)	
>4	36 (36.4%)	31 (64.6%)	
Pathology
Invasive lobular carcinoma	7 (7.1%)	5 (10.4%)	0.345^b^
Invasive ductal carcinoma	92 (92.9%)	43 (89.6%)	
Tumor grade
G1	4 (4.4%)	0 (0%)	0.202^b^
G2	78 (78.8%)	36 (75%)	
G3	17 (17.2%)	11 (22.9%)	
G4	0 (0%)	1 (2.1%)	
Axillary lymph node metastasis
≤4	68 (68.7%)	15 (31.2%)	0.001*^b^
>4	31 (31.3%)	33 (68.8%)	
Lymphovascular invasion
Negative	76 (76.8%)	16 (33.3%)	0.001*^b^
Positive	20 (20.2%)	32 (66.7%)	
NA	3 (3%)	0 (0%)	
ER
Negative	42 (42.4%)	23 (47.9%)	0.083^b^
Positive	47 (47.5%)	15 (31.3%)	
NA	10 (10.1%)	10 (20.8%)	
PR
Negative	46 (46.5%)	23 (47.9%)	0.138^b^
Positive	43 (43.4%)	15 (31.3%)	
NA	10 (10.1%)	10 (20.8%)	
Her-2
Negative	51 (51.5%)	24 (50%)	0.171^b^
Positive	38 (38.4%)	14 (29.2%)	
NA	10 (10.1%)	10 (20.8%)	

*Data are reported as means ± SD*.

*^a^Student’s *t*-test*.

*^b^Fisher’s exact test*.

*Significant *p* value (*p * < 0.05).

*NA = not available*.

### gB-1 and gN-1 are the most prevalent HCMV genotypes found in breast cancer patients

**Figure 1:**
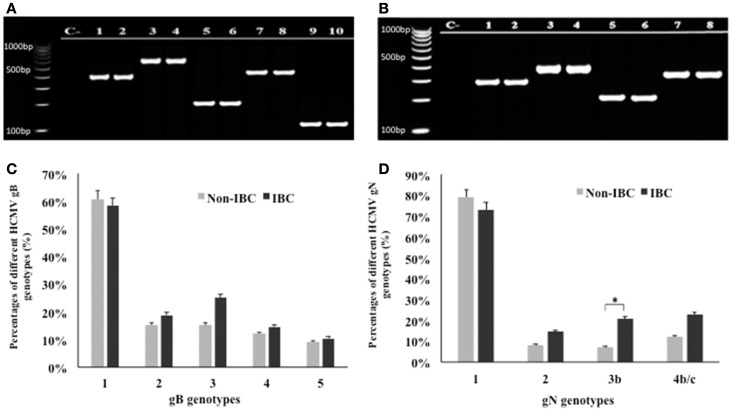
**Distribution frequencies of HCMV genotypes in non-IBC and IBC carcinoma tissues are shown**. **(A)** Agarose gel electrophoresis for nested multiplex PCR showed single detection of different HCMV gB genotypes. Lane C− represents negative control; lanes 1 and 2 represent gB-1 (420 bp) in non-IBC and IBC, respectively. Lanes 3 and 4 represent gB-2 (613 bp) in non-IBC and IBC, respectively. Lanes 5 and 6 represent gB-3 (190 bp) in non-IBC and IBC, respectively. Lanes 7 and 8 represent gB-4 (465 bp) in non-IBC and IBC, respectively, and lanes 9 and 10 represent gB-5 (139 bp) in non-IBC and IBC, respectively. **(B)** Agarose gel electrophoresis for multiplex PCR showed single detection of different HCMV gN genotypes. Lane C− represents negative control; lanes 1 and 2 represent gN-1 (283 bp) in non-IBC and IBC, respectively. Lanes 3 and 4 represent gN-2 (380 bp) in non-IBC and IBC, respectively. Lanes 5 and 6 represent gN-3b (214 bp), in non-IBC and IBC, respectively, and lanes 7 and 8 represent gN-4b/c (244 bp) in non-IBC and IBC, respectively. **(C)** Bars represent distribution frequencies of single infection with different HCMV gB genotypes in non-IBC versus IBC carcinoma tissues. **(D)** Bars represent distribution frequencies of single infection with different HCMV gN genotypes non-IBC versus IBC carcinoma tissues. *Indicates a significant *p* value as determined by Fisher’s exact test.

Application of multiplex PCR for genotyping of HCMV/gB and gN in breast carcinoma tissue samples is shown in Figures 1A,B. Statistical analysis revealed that gB was detected in 95.9% of all carcinoma tissue samples. In non-IBC breast carcinoma tissues, gB-1 was the most prominent genotype representing 60.6% of all infected carcinoma tissues, while gB-2, gB-3, gB-4, and gB-5 represents 15.2, 15.2, 12.1, and 9.1%, respectively. Similarly, in IBC carcinoma tissues, gB-1 was the most prominent genotype, representing 58.3% of all positive carcinoma tissues, while gB-2, gB-3, gB-4, and gB-5 represent 18.8, 25, 14.6, and 10.4%, respectively (Figure 1C).

Glycoprotein N genotyping revealed that gN was detected in 97.45% of all carcinoma tissue samples. In non-IBC carcinoma tissues, gN-1 was the most dominant genotype, representing 78.8% of all infected carcinoma tissues. gN-2, gN-3b, and gN-4b/c represent 8.1, 7.1, and 12.1%, respectively. Similarly, in IBC carcinoma tissues, gN-1 was the most prevalent genotype, representing 72.9% of all positive carcinoma tissues. The incidence of gN-2, gN-3b, and gN-4b/c represent 14.6, 20.8, and 22.9%, respectively. The incidence of gN-3b was significantly higher (*p* = 0.049) in IBC carcinoma tissues compared to non-IBC tissues (Figure 1D).

### Prevalence of mixed HCMV genotypes is predominant among IBC carcinoma tissues compared to non-IBC tissues

**Figure 2:**
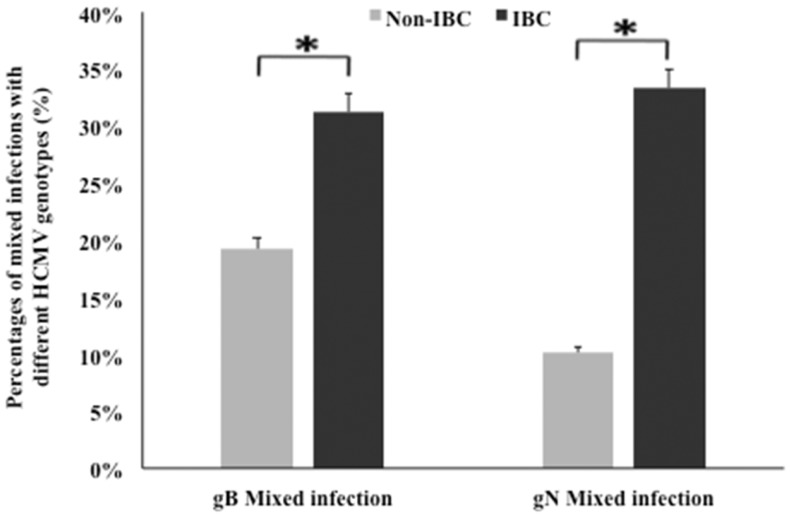
**Incidence of detection of mixed HCMV genotypes in non-IBC and IBC carcinoma tissues is shown**. Bars represent the incidence of mixed presence of different HCMV gB and gN genotypes in non-IBC and IBC carcinoma tissues as detected by multiplex PCR IBC carcinoma tissues characterized by high incidence of mixed HCMV genotypes compared to non-IBC carcinoma tissues.*Indicates a significant *p* value as determined by Fisher’s exact test.

We compared the incidence of detection of HCMV gB and gN genotypes DNA in non-IBC and IBC carcinoma tissues. Our results showed that mixed incidence of gB and gN genotypes was significantly higher (*p* = 0.048 and 0.002, respectively) in IBC carcinoma tissues compared to non-IBC tissues (Figure 2).

**Figure 3:**
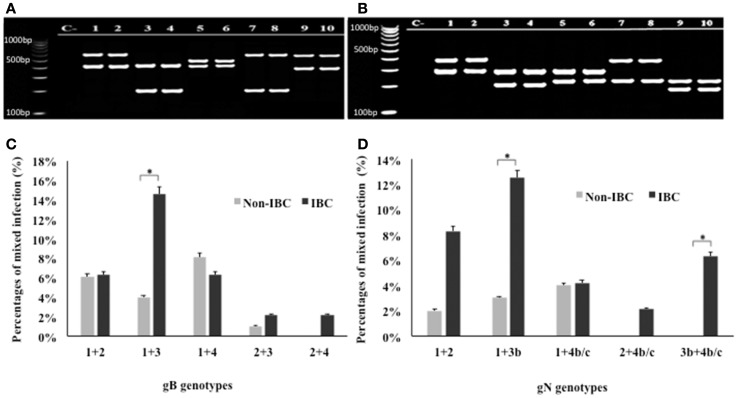
**Incidence of detection of mixed HCMV different gB and gN genotypes in non-IBC and IBC carcinoma tissues is shown**. **(A)** Agarose gel electrophoresis for nested multiplex PCR showed mixed presence of different HCMV gB genotypes. Lane C− represents negative control; lanes 1 and 2 represent gB-1 + gB-2 (420 bp + 613 bp) in non-IBC and IBC, respectively. Lanes 3 and 4 represent gB-1 + gB-3 (420 bp + 190 bp) non-IBC and IBC, respectively. Lanes 5 and 6 represent gB-1 + gB-4 (420 bp + 465 bp) in non-IBC and IBC, respectively. Lanes 7 and 8 represent gB-2 + gB-3 (613 bp + 190 bp) in non-IBC and IBC, respectively. Lanes 9 and 10 represent gB-2 + gB-4 (613 bp + 465 bp) in non-IBC and IBC, respectively. **(B)** Agarose gel electrophoresis for multiplex PCR showed mixed infection with different HCMV gN genotypes. Lane C− represents negative control, lanes 1 and 2 represent gN-1 + gN-2 (283 bp + 380 bp) in non-IBC and IBC, respectively, lanes 3 and 4 represent gN-1 + gN-3b (283 bp + 214 bp) in non-IBC and IBC, respectively, lanes 5 and 6 represent gN-1 + gN-4b/c (283 bp + 244 bp) in non-IBC and IBC, respectively, lanes 7 and 8 represent gN-2 + gN-4b/c (380 bp + 244 bp) in non-IBC and IBC, respectively, and lanes 9 and 10 represent gN-3b + gN-4b/c (214 bp + 244 bp) in non-IBC and IBC, respectively. **(C)** Bars represent the incidence of mixed infection with different HCMV gB genotypes in non-IBC and IBC carcinoma tissues as detected by multiplex PCR. **(D)** Bars represent the incidence of mixed infection with different HCMV gN genotypes. Non-IBC bar represents the percentage of mixed gN genotypes infections in non-IBC and IBC carcinoma tissues as detected by multiplex PCR. *Indicates a significant *p* value as determined by Fisher’s exact test.

Detection of mixed genotypes of HCMV in non-IBC and IBC carcinoma tissues was assessed by multiplex PCR (Figures 3A,B). Statistical analysis revealed that in non-IBC carcinoma tissues, mixed infection with gB-1 + gB-4, gB-1 + gB-2, gB-1 + gB-3, and gB-2 + gB-3 represents 8.1, 6.1, 4, and 1%, respectively. In IBC carcinoma tissues, we found that mixed infection with gB-1 + gB-3, gB-1 + gB-2, gB-1 + gB-4, gB-2 + gB-3, and gB-2 + gB-4 represents 14.6, 6.3, 6.3, 2.1, and 2.1%, respectively (Figure 3C). Mixed gB-2 + gB-4 genotypes could not be detected in non-IBC carcinoma tissues. Detection of mixed gB-1 + gB-3 genotypes was significantly higher (*p* = 0.029) in IBC carcinoma tissue than non-IBC tissues. Distribution of the detected mixed gN genotypes revealed that in non-IBC carcinoma tissues, gN-1 + gN-4b/c, gN-1 + gN-2, and gN-1 + gN-3b represent 4, 2, and 3%, respectively. In IBC carcinoma tissues, gN-1 + gN-3b, gN-1 + gN-2, gN-1 + gN-4b/c, gN-2 + gN-4b/c, and gN-3b + gN-4b/c represent 12.5, 8.3, 4.2, 2.1, and 6.3%, respectively (Figure 3D). Detection of mixed gN-1 + gN-3b genotypes and gN-3b + gN-4b/c genotypes was significantly higher (*p* = 0.034 and 0.033, respectively) in IBC patient tissues than non-IBC tissues.

To validate these results, we conducted DNA sequence analysis of whole genomes of gB and gN, and the entire gene sequences of different gB and gN genotypes compared to those represented in GenBank. The accession numbers KJ778194, KJ778196, KJ778198, KJ778200, and KJ778202 represent the entire gB genotype (g1, g2, g3, g4, and g5, respectively) sequences obtained from non-IBC carcinoma tissues. The accession numbers KJ778193, KJ778195, KJ778197, KJ778199, and KJ778201 represent the entire gB genotypes sequence (gB-1, gB-2, gB-3, gB-4, and gB-5) sequences obtained from IBC carcinoma tissues. The sequence of gN had the accession numbers KF875977, KF875979, KF875981, and KF875983, and represents the entire gN genotype (gN-1, gN-2, gN-3b, and gN-1–4b/c) sequences obtained from non-IBC carcinoma tissues. Accession numbers KF875976, KF875978, KF875980, and KF875982 represent the entire gN genotypes (gN1, gN-2, gN-3b, and gN-4b/c) sequences obtained from IBC carcinoma tissues, respectively.

### Detection of mixed HCMV different genotypes significantly correlates with the number of metastatic lymph nodes in non-IBC

**Figure 4:**
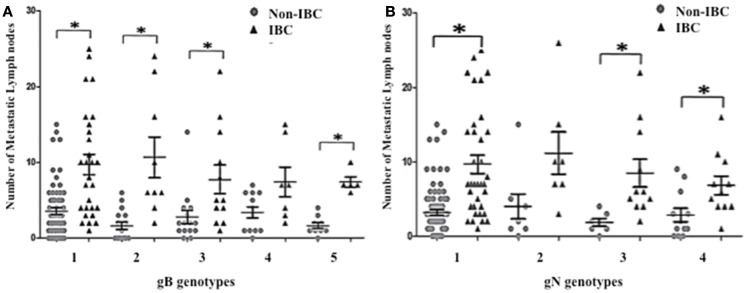
**Number of positive metastatic lymph nodes among HCMV positive non-IBC and IBC patients by HCMV gB or gN genotypes is shown**. **(A)** Dots represent the numbers of metastatic lymph nodes in non-IBC and IBC patients containing different HCMV gB genotypes. **(B)** Dots represent the numbers of metastatic lymph nodes in non-IBC and IBC patients containing different HCMV gN genotypes. *Indicates a significant *p* value as determined by Student’s *T*-test.

**Figure 5:**
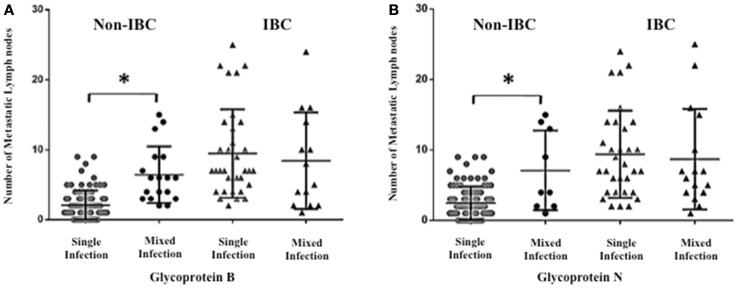
**Association between lymph node metastasis and mixed presence of HCMV genotypes in IBC and non-IBC patients is shown**. **(A)** Dots represent the numbers of metastatic lymph nodes in non-IBC and IBC patients containing mixed HCMV gB genotypes. **(B)** Dots represent the numbers of metastatic lymph nodes in non-IBC and IBC patients with mixed HCMV gN genotypes. Results showed that detection of mixed HCMV different gB or gN genotypes statistically correlates with lymph node metastasis in non-IBC. *Indicates a significant *p* value as determined by Pearson’s correlation coefficient.

To investigate whether detection of mixed HCMV genotypes is associated with the metastatic behavior of breast carcinoma cells, we assessed the distribution of the number of metastatic lymph nodes among non-IBC and IBC patients with different gB and gN genotypes. The number of metastatic lymph nodes in gB-1, gB-2, gB-3, and gB-5 was significantly higher (*p* = 0.001, 0.009, 0.031, and 0.001, respectively) in IBC than non-IBC patients (Figure 4A). In addition, the number of metastatic lymph nodes in gN-1, gN-3b, and gN-4b/c was significantly (*p* = 0.001, 0.006, and 0.019) higher in IBC than non-IBC patients (Figure 4B). Interestingly, detection of mixed HCMV different gB genotypes (Figure 5A) and gN genotypes (Figure 5B) was statistically correlated with the number of metastatic lymph nodes in non-IBC (*p* = 0.001 and 0.001, respectively) with a correlation coefficient of *r* = 0.550 and 0.437, respectively, and not in IBC.

### Detection of mixed HCMV different genotypes significantly correlates with lymphovascular invasion and formation of dermal lymphatic emboli in IBC

**Figure 6:**
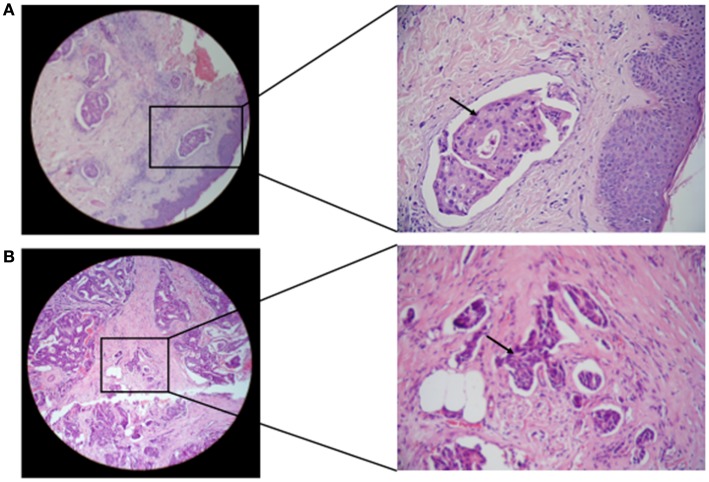
**Microscopic images of H and E stained paraffin embedded tissue sections in breast carcinoma tissues are shown**. **(A)** Representative of paraffin embedded tissue section of non-IBC carcinoma tissues showing invasion of carcinoma cells to lymphatic vessels. **(B)** Representative of paraffin-embedded tissue section of IBC carcinoma tissues showing tumor emboli formation due to carcinoma cells invasion into lymphatic vessels (magnification: left panel, 10× and right panel, 40×).

The status of lymphovascular invasion and formation of dermal lymphatic emboli (Figure 6) among carcinoma tissues containing different gB and gN genotypes is described in Table 3. Patients showing gB-1, gB-2, gB-3, and gB-5 were significantly (*p* = 0.001, 0.016, 0.002, and 0.032, respectively) higher in IBC than non-IBC patients. gN-1, gN-3b, and gN-4b/c were significantly (*p* = 0.001, 0.036, and 0.008, respectively) higher in IBC than non-IBC patients. Detection of mixed HCMV different gB or gN genotypes was found to be significantly correlated (*p* = 0.037 or 0.021, respectively) with lymphovascular invasion status in IBC. In addition, detection of mixed HCMV different gB or gN genotypes was statistically correlated with formation of dermal lymphatic emboli in IBC (*p* = 0.01 or 0.004, respectively) and correlation coefficient *r* = 0.368 and 0.408, respectively.

**Table 3:** **Incidence of lymphovascular invasion status among different gB and gN genotypes in non-IBC versus IBC patients**.

HCMV glycoproteins	Non-IBC lymphovascular invasion status		IBC lymphovascular tumor emboli		*p* Value
	Positive	Negative	Positive	Negative	
**gB GENOTYPES**					
gB-1	28.3% (*n *= 17)	71.7% (*n *= 43)	75% (*n *= 21)	25% (*n *= 7)	0.001*
gB-2	25% (*n *= 4)	75% (*n *= 12)	77.8% (*n *= 7)	22.2% (*n *= 2)	0.016*
gB-3	6.7% (*n *= 1)	93.3% (*n *= 14)	66.7% (*n *= 8)	33.3% (*n *= 4)	0.002*
gB-4	36.4% (*n *= 4)	63.6% (*n *= 7)	85.7% (*n *= 6)	14.3% (*n *= 1)	0.057
gB-5	12.5% (*n *= 1)	87.5% (*n *= 7)	80% (*n *= 4)	20% (*n *= 1)	0.032*
**gN GENOTYPES**					
gN-1	21.8% (*n *= 17)	78.2% (*n *= 61)	74.3% (*n *= 26)	25.7% (*n *= 9)	0.001*
gN-2	37.5% (*n *= 1)	62.5% (*n *= 7)	57.1% (*n *= 4)	42.9% (*n *= 3)	0.100
gN-3b	14.3% (*n *= 1)	85.7% (*n *= 6)	70% (*n *= 7)	30% (*n *= 3)	0.006*
gN-4b/c	8.3% (*n *= 1)	91.7% (*n *= 11)	63.6% (*n *= 7)	36.4% (*n *= 4)	0.008*

*Significant *p* value (*p* < 0.05).

## Discussion

There is emerging evidence to suspect that viruses may play a role in the development or progression of certain human cancers, possibly including breast cancer (47). Using nested PCR, our previous results revealed that HCMV-DNA was detected in carcinoma tissues of IBC and not in adjacent non-carcinoma tissues, with results that are statistically significant compared to non-IBC patients’ group. Interestingly, sequence analysis of the detected HCMV-DNA fragments revealed that HCMV-infected IBC carcinoma tissues contain different HCMV-strains when compared to infected non-IBC tissues. Polymorphism among HCMV strains may provide important clinical information on the involvement of HCMV in IBC disease etiology (43). In fact, studies showed that HCMV is characterized by genetic variability among its strains, which plays an important role in immune-pathogenesis and stimulation of disease progression. In addition, the ability of HCMV to infect different organs and cell types was found to be related to gene sequence variation among strains (14, 48, 49). Enhancement of CMV disease pathogenesis due to mixed strain infection may result in expression and secretion of cytokines, chemokines, and growth factors due to replication of different virus strains (50). Indeed, cytokines, chemokines, and growth factors secreted by breast carcinoma cells and tumor-associated immune cells such as monocytes/macrophages play an essential role in breast cancer progression (51), and may provide therapeutic target molecules (42, 52).

The genomes of different HCMV strains are 95% homologous, but specific regions contain higher rates of mutation (53). The most widely characterized polymorphic gene is *UL55*, which encodes the viral gB genes, which is essential for virus penetration and cell fusion (54), and represents the major target for neutralizing antibodies (55). The *UL73* gene, which encodes viral gN implicated in virus attachment to the host cell and spread from cell to cell, is another highly polymorphic gene (56).

In the present study, we analyzed the incidence of gB and gN genotypes in carcinoma tissues of breast cancer patients and its association with breast cancer disease progression. Multiplex PCR revealed that gB-1 is the most prevalent genotype in both non-IBC and IBC carcinoma tissues with no significant difference between both patient groups. The present result is in agreement with previous studies, which reported that gB-1 had the highest incidence in renal transplant recipient HCMV-infected patients in Kuwait (57) and among children who live at the Phayathai Babies’ Home in Non-thaburi, Thailand (58). The predominance of the gB-1 genotype in HCMV-infected patients was also recorded in immunocompromised patients undergoing organ transplants (15) and congenitally infected neonates (59, 60) in a Chinese population. The gB-1 genotype was found to be the dominant HCMV genotype detected in pregnant women, newborns, and infants. The previous results increase the possibility that gB-1 might be transmitted by breast feeding (58, 61, 62), and is more frequent in women of different populations.

Similarly, we found that gN-1 is the most prominent genotype in both non-IBC and IBC carcinoma tissues with no significant difference between both patient groups. HCMV gN-1 was the most incident genotype in monocytes isolated from healthy blood donors (21). In this regard, HCMV gN-1 congenital infections are associated with favorable chronic outcome (19). In the present study, we found that IBC carcinoma tissues showed a statistically significantly higher incidence of the gN-3b genotype compared to non-IBC tissues.

Detection of mixed HCMV genotypes gB and gN was found in urine and peripheral blood of healthy women (63) and in vaginal specimens of women with sexually transmitted diseases (64). Immunocompromised individuals acquire mixed infection of HCMV genotypes (15, 33). Infection with mixed HCMV genotypes was found to enhance immunopathogenesis (18) and morbidity (65) among infected patients. In the present study, we found that detection of mixed HCMV gB and gN genotypes were more prominent in carcinoma tissues of IBC versus non-IBC patients. Distribution of mixed presence of different gB genotypes showed that gB-1 + gB-3 was statistically significantly higher in IBC than non-IBC carcinoma tissues. IBC carcinoma tissues are characterized by highly invasive and angiogenic properties (66). Studies showed that gB-1 is the most frequent genotype among kidney transplant patients and induces the progression of invasive disease in solid organ transplants (67). Other studies showed that genotypes gB-1 and gB-3 are more prevalent among hematopoietic stem cell transplant (HSCT) patients and that gB-3 is associated with the development of pneumonitis (68). A recent study showed that HSCT patients presenting with the gB-3 genotype are characterized by higher disease morbidity and lower survival rates compared to patients harboring genotypes gB-1, -2, and -4 (69). The aggressive behavior of genotype gB-3 may be due to specific biological mechanisms associated with host–virus interactions (70). Our present results suggest that the presence of HCMV-gB-3 aggressive genotype among IBC patients might affect IBC poor prognosis and disease morbidity. In the present study, we found that detection of mixed HCMV gN genotypes in breast carcinoma tissues showed that gN-1 + gN-3b and gN-3 + gN-4b/c were statistically significantly higher in IBC than non-IBC carcinoma tissues. In particular, previous studies suggested that HCMV may have a “strain-specific pathogenic phenotype,” which may be due to a link conferred by polymorphic gN genes and their function together (18, 71, 72).

Prospective survival rates among breast cancer patients are determined by the potential of carcinoma cells to invade lymph nodes and the number of metastatic axillary lymph nodes involved (73). A study conducted by Soderberg-Naucler showed that HCMV genes and proteins are present in the carcinoma tissues and the sentinel lymph nodes of metastatic breast cancer patients (74). An interesting study in cervical cancer showed that patients co-infected with both HCMV and HPV 16 had a higher number of metastatic lymph nodes compared to HCMV^−^/HPV^+^ (75). Indeed, HCMV-infected cells secrete cytokines and growth factors (secretome) that promote cellular motility, angiogenesis, and lymphangiogenesis (12, 76). Over expression of US28 encoded by HCMV is strongly associated with glioblastoma multiforme tumor invasion, possibly via activation of VEGF, phosphorylation of STAT3 (*p*-STAT3), and endothelial nitric oxide synthase (e-NOS) (77).

In the present study, we found that the number of metastatic lymph nodes in patients with breast carcinoma tissues containing HCMV gB-1, gB-2, gB-3, and gB-5 was significantly higher in IBC than non-IBC patients. Similarly, the number of metastatic lymph nodes in gN-1, gN-3b, and gN-4b/c was statistically significantly higher in IBC patients. IBC patients were characterized by a statistically significantly higher number of axillary metastatic lymph nodes compared to non-IBC patients. The novel results we found here, that mixed infections with different gB or gN genotypes significantly correlates with number of metastatic lymph nodes in non-IBC patients, possibly indicates that mixed infection with HCMV genotypes may augment invasion and motility of breast carcinoma cells.

Lymphovascular invasion is an important step in breast cancer metastasis, with formation of lymphovascular emboli in IBC (78). IBC is an aggressive form of breast cancer, characterized by formation of lymphatic emboli within the lymphatic vessels (79). HCMV infection in tumor cells increases their adhesion to endothelium by activation of β1α5 integrin on the surface of infected tumor cells, which promotes tumor cell transmigration through the endothelial cell barrier. The HCMV US28 pathway contributes to FAK activation and stimulation of cell invasion in HCMV-infected tumor cells (80, 81). Similar to results of lymph node metastasis, lymphovascular invasion in patients demonstrating breast carcinoma tissues with gB-1, gB-2, gB-3, and gB-5 genotypes was statistically significantly higher in IBC than non-IBC tissues. In the breast carcinoma tissues with single gN genotype, we found that lymph node metastasis in gN-1, gN-3b, and gN-4b/c genotypes was statically significantly higher in IBC than non-IBC patients. Detection of mixed HCMV different genotypes significantly correlates with lymphovascular invasion and formation of dermal lymphatic emboli in IBC but not in non-IBC patients.

In conclusion, our present results suggest a possibility of a high incidence of mixed HCMV genotypes infection in IBC, which may play a role in IBC disease progression. Further studies are necessary to determine the clinical significance of our findings and potential treatment of HCMV-infected breast cancer patients.

## Conflict of Interest Statement

The authors declare that the research was conducted in the absence of any commercial or financial relationships that could be construed as a potential conflict of interest.
